# Prevalence of Trachoma after Implementation of Trachoma Elimination Interventions in Oromia Regional State, Ethiopia: Results of Impact Surveys in 131 Evaluation Units Covering 139 Districts

**DOI:** 10.1080/09286586.2022.2119257

**Published:** 2022-12-15

**Authors:** Hirpa Miecha, Michael Dejene, Dereje Adugna, Ageru Kebede, Damtew Yadeta, Addisu Alemayehu, Aemero Abateneh, Asfaw Wondimu, Mihiret Dayessa, Muhammed Shafi, Emawayish Taye, Leta Balcha, Solomon Gadisa, Nebiyu Negussu, Belete Mengistu, Rebecca Willis, Cristina Jimenez, Ana Bakhtiari, Sarah Boyd, Biruk Kebede, Fantahun Tadesse, Ayele Mamo, Mengistu Bekele, Zelalem Sinke, Anthony W. Solomon, Emma M. Harding-Esch

**Affiliations:** a Oromia Regional Health Bureau, Addis Ababa, Ethiopia; b Sightsavers, Addis Ababa, Ethiopia; c The Fred Hollows Foundation, Addis Ababa, Ethiopia; d Research Triangle Institute, Addis Ababa, Ethiopia; e Jimma University, Jimma, Ethiopia; f Asfaw Wondimu Health Research and Consultancy, Addis Ababa, Ethiopia; g Ambo Hospital, Ambo, Ethiopia; h Adama Hospital Medical College, Adama, Ethiopia; i Light For The World, Addis Ababa, Ethiopia; j Federal Ministry of Health, Addis Ababa, Ethiopia; k Department of Control of Neglected Tropical Diseases, World Health Organization, Geneva, Switzerland; l International Trachoma Initiative, Task Force for Global Health, Decatur, Georgia, USA; m Clinical Research Department, London School of Hygiene & Tropical Medicine, London, UK

**Keywords:** trachoma, blindness, trichiasis, prevalence

## Abstract

**Purpose:**

To determine the prevalence of trachomatous inflammation—follicular (TF), trachomatous trichiasis (TT), water, sanitation, and hygiene (WASH) access in 131 evaluation units (EUs) after implementation of trachoma elimination interventions in Oromia Region, Ethiopia.

**Methodology:**

A population-based cross-sectional survey was conducted in each EU using the World Health Organization-recommended two-stage cluster-sampling methodology. Twenty-six clusters, each with a mean of 30 households were enumerated in each EU. All residents aged ≥1 year in selected households were examined for TF and TT. Information on WASH access in surveyed households was also collected through questioning the household head and direct observation.

**Results:**

A total of 419,858 individuals were enumerated in 131 EUs, of whom 396,134 (94%) were examined, 54% being female. Age-adjusted EU-level prevalence of TF in children aged 1–9 years ranged from 0.15% (95% confidence interval [CI]: 0.0–0.4) to 37.5% (95% CI: 31.1–43.7). The TF prevalence was <5% in 73/131 (56%) EUs. The EU-level age- and gender-adjusted prevalence of TT unknown to the health system among people aged ≥15 years ranged from 0.001% (95% CI: 0.00–0.02) to 2.2% (95% CI: 1.1–3.1) with 37/131 (28%) EUs having a prevalence <0.2%. Only 48% of all households surveyed had access to improved water sources for drinking. Approximately 96% of households did not have an improved latrine.

**Conclusion:**

Oromia is on the path towards elimination of trachoma as a public health problem.

## Introduction

Trachoma is a neglected tropical disease and the leading infectious cause of blindness.^1^ The disease is caused by certain serovars of the bacterium *Chlamydia trachomatis*, which are transmitted within ocular and nasal secretions passed from person to person on fingers, fomites (such as clothing), and eye-seeking flies (particularly *Musca sorbens*).^2–6^ Global efforts are underway to eliminate trachoma as a public health problem.^7–9^ The World Health Organization (WHO)-recommended strategy to achieve elimination is known as SAFE (surgery, antibiotics, facial cleanliness, and environmental improvement).^10^ An EU is a district that for the purposes of trachoma elimination WHO defines as “the normal administrative unit for health care management, consisting of a population unit between 100,000–250,000 persons”.^11^

WHO estimates indicated that 166.6 million people lived in 1437 districts of 37 countries in which the trachomatous inflammation—follicular (TF) prevalence in children aged 1–9 years was ≥5% at some time during 2019.^12^ About 87% (144.6 million) of these individuals were in WHO’s African Region, 76.2 million (46% of the global total) of whom were in Ethiopia.^12^ Within Ethiopia, 673 districts (about 85% of all districts) were in need of treatment with antibiotics, facial cleanliness, and environmental improvement interventions for the elimination of active trachoma as a public health problem.

With an estimated population of over 39 million people, Oromia is divided into 19 town administration units and 21 rural zones; with 291 rural and 46 urban woredas. (For the purposes of this manuscript, a woreda is equivalent to a district; the term district will be used throughout.)

A total of 252 districts grouped into 79 sub-zones (EUs) in the Oromia region were mapped from 20122014 as part of the Global Trachoma Mapping Project (GTMP).^13–15^ Fifteen EUs had a TF prevalence <5%, 8 EUs a prevalence 5–9.9%, 26 EUs a prevalence 10–29.9% and 30 EUs a prevalence ≥30%, resulting in 64 EUs covering 232 districts requiring “AFE” interventions before impact surveys could be conducted. It was further noted that 72 EUs covering 240 districts had trichiasis prevalence of ≥0.2% in those aged ≥15 years, requiring public health-level TT surgery services to be implemented. From this, an estimate of about 98,000 cases were waiting for corrective surgery as part of the trachoma elimination strategy.

Since 2013, SAFE interventions have been scaled up in districts that require them and the region achieved 100% geographic coverage in 2019. All districts covered by the baseline surveys have completed the number of annual rounds of antibiotic mass drug administration (MDA) recommended by WHO before implementing a trachoma impact survey.^16^ Accordingly, all districts have received 1–5 rounds of MDA with an average of above 80% therapeutic coverage. Clean water source development to increase access to safe water for drinking and personal hygiene was undertaken in addition to latrine construction. Awareness creation about the importance of face washing, hand hygiene and use of improved sanitation facilities has also been carried out.^17^

Re-establishing the current burden of disease in Oromia region is important to help determine if interventions have achieved the trachoma elimination thresholds. We present TF and TT prevalence and associated water, sanitation, and hygiene data from impact surveys conducted between January 2017 and March 2020 in 139 districts.

## Methods

### Ethics and consent

Prior to survey commencement, ethical clearance was obtained from the Oromia Regional Health Bureau ethical clearance committee (BEFO/AHDFIDh/1-69/3079). Tropical Data survey support was approved by the London School of Hygiene & Tropical Medicine Observational Ethics Committee (16105).

Before participating, each person was informed about the objectives of the work and gave verbal consent to take part. Parents, a guardian, or the household head verbally consented on behalf of children aged <18 years, who are not legally entitled to give consent.

People identified as having active trachoma (TF and/or trachomatous inflammation—intense [TI]) or any other likely-bacterial eye condition were provided with 1% tetracycline eye ointment with instructions for its use, while people identified as having TT were referred to the nearest health facility.

### Study design, participant selection

While this survey series was being implemented, WHO updated its definition of TT based on discussions at the fourth Global Scientific Meeting on Trachoma (GSM4), held in November 2018.^18^ These changes, later integrated into the WHO simplified grading system,^19^ were incorporated into Tropical Data protocols. In the pre-GSM4 surveys, TT was defined as any eyelash touching the eyeball or evidence of recent removal of in-turned eyelashes, whereas post-GSM4, TT was defined as any eyelash from the upper eyelid touching the eyeball, or evidence of recent removal of in-turned eyelashes from the upper eyelid. At the same time, Tropical Data also updated the WASH questionnaire to reflect the latest WHO/UNICEF Joint Monitoring Programme (JMP) for Water Supply, Sanitation, and Hygiene core questions for households.^20^ This included a change from handwashing facilities only being noted if they were within 15 m of the latrine (pre-GSM4), to determining whether there was a handwashing facility in the yard/plot/premises (post-GSM4); data on hand washing facilities therefore began to be recorded for all households as the question was no longer dependent on the household having a latrine.^21^

We used a community-based cross-sectional survey design.^15^ Most EUs consisted of a single district. Some pairs of adjacent districts with small populations were combined into a single EU. There were 139 EUs that completed the recommended number of MDA rounds in the time period covered by this survey series and, of those, impact surveys were carried out in 131 EUs (incorporating 139 districts). Surveys were not conducted in 8 EUs due to persistent security issues.

In each EU, a two-stage cluster sampling technique was employed to select participants. During the first stage of sampling, systematic selection with probability proportional to size was used to select 26 primary sampling units (referred to as clusters in this manuscript and defined for these surveys as a gare, a sub-unit of a village. In the second stage of sampling, compact segment sampling was employed to select 30 households, the secondary sampling unit, from each selected gare.

A structured questionnaire was administered to the head of each selected household (or another adult member of the household) to assess household access to WASH facilities. All residents of the household aged ≥1 year were then invited to be examined for clinical signs of trachoma.

### Sampling and sample size

Sample size was estimated using the single population proportion for precision formula, and in 8 EUs with population<100,000 the sample size was adjusted to correct for a small, finite population.^22,23^ All surveys were designed to estimate TF prevalence of 4% in 1–9-year-olds with a precision of ±2% at the 95% confidence level.^23^ Assuming a design effect of 2.63^24^ and using a non-response inflation factor of 1.2, it was determined that for each EU with population ≥100,000, 1,164 children aged 1–9 years would need to be enumerated.

For all EUs, individuals ≥15 years of age who lived in households selected to survey children formed the sample for estimating TT prevalence. The loss of precision resulting from this approach was accepted.^15,23,25^

### Training of survey team

The training of graders and recorders was conducted as per the training system outlined in the relevant Tropical Data training manuals (version 2 for surveys undertaken pre-GSM4^26^; version 3 for surveys undertaken post-GSM4).^21^

### Data collection

All data were captured electronically using the purpose-built Open Data Kit-based Tropical Data Android smartphone application.

Each individual who consented for the study was examined using 2.5× magnifying binocular loupes, and sunlight or a torch, by certified graders for TF, TI, and trichiasis. In post-GSM4 surveys, follicle size guides were used to aid the diagnosis of TF,^27^ and both upper and lower eyelid trichiasis was recorded separately. Where trichiasis was recorded as being present in an eye, the presence or absence of conjunctival trachomatous scarring (TS) was assessed and the individual was questioned about whether they had been offered management for trichiasis, and if they had, whether they had accepted the offer. If one or more resident 1–9-year-olds was missing at the time of the first visit, the household was re-visited before the end of the day to increase response rates.

### Data analysis

Analysis of TF and TT prevalence, including adjustments for age in one-year bands, and for age and gender in five-year bands, respectively, were conducted as described elsewhere.^15^ TF prevalence was directly compared between pre-MDA surveys and the survey data presented here without further adjustment or extrapolation to account for the difference in age and gender of the populations surveyed. The EU boundaries and sampling frames in pre-MDA surveys often encompassed several districts (parent EU), whereas in the impact surveys presented here the EU boundaries and sampling frames mostly comprised a single district (offspring EU). This led to multiple offspring EUs from a single-parent EU and prevents meaningful comparison between pre- and post-MDA prevalence estimates. The TF and TT prevalence estimates are therefore presented here at face value and no analyses for statistically significant changes over time have been carried out.

## Results

Of 419,858 individuals enumerated, 396,134 (94.3%) consenting individuals were examined across the 131 EUs (Table 1). Totally, 22,855 individuals were absent from their household on the day of the household visit and 861 refused to participate.

**Table 1.** Protocol employed, population enumerated and participants examined in trachoma impact surveys, Oromia Region, Ethiopia, February 2017− March 2020. Individual evaluation unit (EU)-level data are shown in Supplementary Table 1.

Zone	EUs surveyed	Survey protocol		Participants enumerated (≥1 years) per EU			Participants examined (≥1 years) per EU		
		Pre-GSM4^a^	Post-GSM4^b^	Minimum	Median	Maximum	Minimum	Median	Maximum
Arsi	25	0	25	2,681	2,994	3,229	2,626	2,856	3,065
Borena	11	11	0	2,539	2,810	3,147	2,459	2,701	3,013
Buno Bedele	6	6	0	3,281	3435	3,538	2,997	3,172	3,270
East Harerge	3	3	0	3,119	3,130	3,475	2,894	2,943	3,282
East Shewa	4	3	1	2,835	3,092	3,453	2,707	2,875	3,152
East Welega	10	10	0	3,334	3,429	3,632	3,106	3,218	3,443
Finfine Zuriya	1	1	0	3,334	3,334	3,334	3,006	3,006	3,006
Guji	5	5	0	3,108	3,229	3,272	3,027	3,153	3,187
Illu Aba bora	9	9	0	3,222	3,339	3,818	2,990	3,108	3,551
Jimma	9	3	6	2,931	3,034	3,709	2,694	2,960	3,458
Kelem Welega	3	3	0	3,296	3,364	3,390	3,121	3,159	3,223
North Shoa	9	9	0	3,110	3,385	3,650	2,941	3,081	3,280
South West Shewa	11	0	11	2,943	3,043	3,268	2,780	2,909	3,342
West Shewa	12	12	0	3,248	3,539	3,666	2,990	3,334	3,473
West Arsi	10	2	8	2,871	3,278	3,559	2,679	3,084	3,330
West Welega	3	3	0	3,074	3,229	3,381	2,957	3,092	3,234
Total	131	80	51	2,681	3,222	3,818	2,459	3,006	3,551

^a^Pre-4^th^ Global Scientific Meeting on Trachoma (GSM4): Tropical Data survey version 2; ^b^Post-GSM4: Tropical Data survey version 3.

### Prevalence of trachomatous inflammation—follicular

Among 142,665 1–9-year-olds examined, 10,298 (7.2%) had TF in one or both eyes. Considerable variation in the age-adjusted EU-level TF prevalence among 1−9-year-olds was observed: it ranged from 0.2% (95% confidence interval [CI]: 0.0−0.4) in Yubdo & Homa district of West Welega zone to 37.5% (95% CI: 31.1−43.7) in Derra district of North Shoa zone. Overall, 73 EUs (55.7% of all surveyed EUs) had a TF prevalence in 1–9-year-olds <5%, the WHO elimination target for active trachoma. Of the 58 EUs that had a TF prevalence ≥5%, the prevalence was between 5.0% and 9.9% in 38 EUs (29.0%), between 10.0% and 29.9% in 19 EUs (14.5%) and ≥30% in one EU (0.8%) (Table 2, Supplementary Table 2).

**Table 2.** Age-adjusted prevalence of trachomatous inflammation—follicular (TF) in 1–9-year-olds at trachoma impact surveys in Oromia Region, Ethiopia, February 2017− March 2020. Individual evaluation unit (EU)-level data are shown in Supplementary Table 2.

Zone	1–9-year-olds examined per EU			Number of EUs with age-adjusted prevalence of TF in 1–9-year-olds in each category			
	Minimum	Median	Maximum	<5%	5.0–9.9%	10.0–29.9%	≥30%
Arsi	799	1,013	1,248	16	5	4	0
Borena	1,088	1,186	1,338	6	4	1	0
Buno Bedele	991	1,077	1,292	4	2	0	0
East Harerge	1,123	1,193	1,215	1	0	2	0
East Shewa	885	1,042	1,160	3	1	0	0
East Wellega	873	1,113	1,274	9	1	0	0
Finfine Zuriya	888	888	888	0	0	1	0
Guji	1,375	1,430	1,475	1	3	1	0
Illu Aba bora	679	977	1,072	9	0	0	0
Jimma	944	1,061	1,178	5	4	0	0
Kelm Wellega	877	898	922	3	0	0	0
North Shoa	865	1,092	1,155	0	6	2	1
South West Shewa	911	995	1,124	0	9	2	0
West Shewa	888	1,200	1,298	11	1	0	0
West Arsi	1,087	1,255	1,394	2	2	6	0
West Wellega	842	850	1,149	3	0	0	0
Total	679	1,088	1,475	73	38	19	1

### Prevalence of trachomatous trichiasis

There were 206,718 people aged ≥15 years examined in the 131 EUs. In the 80 pre-GSM4 EUs, 130,153 people aged ≥15 years were examined, of whom 1,003 (0.8%) were found to have TT (upper or lower eyelid trichiasis) unknown to the health system. The age- and gender-adjusted prevalence of TT unknown to the health system among people aged ≥15 years ranged from 0.01 (95% CI: 0.00–0.03) in Didu district of Ilu Aba Bora zone to 1.87% (95% CI: 1.22–2.61) in Derra district of North Shoa zone. Overall, 21 of the 80 pre-GSM4 EUs (26.3%) had a TT prevalence unknown to the health system <0.2% (Supplementary Figure 1, Table 3, Supplementary table 4). Among the 76,565 people aged ≥15 years examined in the 51 post-GSM4 EUs, 705 (0.9%) were found to have TT (upper eyelid trichiasis) unknown to the health system. The age- and gender-adjusted prevalence of TT unknown to the health system among people aged ≥15 years ranged between 0.001% (95% CI: 0.00–0.02) in Kore district of West Arsi zone to 2.2% (95% CI: 1.2–3.1) in Sokoru district of Jimma zone (Supplementary Figure 1, Table 3, Supplementary Table 5).

**Table 3.** Age-and gender-adjusted prevalence of trachomatous trichiasis (TT) unknown to the health system in people aged ≥15 years in Oromia Region, Ethiopia, February 2017− March 2020.

Zone	People aged ≥15 years examined per EU			# of EUs with prevalence of TT (pre-GSM4 definition; upper or lower eyelid) trichiasis) unknown to the health system in ≥15-year-olds in each category			# of EUs with prevalence of TT (post-GSM4 definition; upper eyelid trichiasis only) unknown to the health system in ≥15-year-olds in each category		
	Minimum	Median	Maximum	<0.2	0.2 − 0.99	≥1.0	<0.2	0.2 − 0.99	≥1.0
Arsi	1,341	1,500	1,691	0	0	0	9	14	2
Borena	1,085	1,347	1,390	2	9	0	0	0	0
Buno Bedele	1,568	1,642	1,733	0	6	0	0	0	0
East Harerge	1,400	1,454	1,652	0	3	0	0	0	0
East Shewa	1,369	1,479	1,975	0	3	0	0	1	0
East Wellega	1,624	1,753	1,816	0	8	2	0	0	0
Finfine Zuriya	1,691	1,691	1,691	0	1	0	0	0	0
Guji	1,316	1,373	1,462	4	1	0	0	0	0
Illu Aba bora	1,657	1,786	2,152	6	3	0	0	0	0
Jimma	1,414	1,516	1,913	2	1	0	0	4	2
Kelm Wellega	1,794	1,800	1,858	1	2	0	0	0	0
North Shoa	1,516	1,657	1,734	0	7	2	0	0	0
South W/Shewa	1,398	1,514	1,630	0	0	0	1	7	3
West Shewa	1,603	1,674	1,879	4	8	0	0	0	0
West Arsi	1,341	1,393	1,572	0	1	1	6	2	0
West Wellega	1,745	1,917	1,997	2	1	0	0	0	0
Total	1,085	1,568	2,152	21	54	5	16	28	7

EU: evaluation unit; Pre-4^th^ Global Scientific Meeting on Trachoma (GSM4): Tropical Data survey version 2; post-GSM4: Tropical Data survey version 3.

### Household access to water and sanitation

Of the 103,549 households (median per EU: 781; range: 663–901) surveyed, 67,007 (65%) reported access to drinking water from an improved source, and 49,705 (48%) had access to an improved drinking water source within a 30-minute return journey of the house. The majority of visited households (99,768; 96%) did not have an improved latrine^28^ (Table 4, Supplementary Table 5).

**Table 4.** Zone-level summaries of proportion of households surveyed with access to improved drinking water facilities and latrines in 131 trachoma impact surveys in Oromia Region, Ethiopia, February 2017− March 2020. Individual evaluation unit (EU)-level data are shown in Supplementary Table 5.

Zone	Per-EU proportion of households with an improved drinking water source (%)			Per-EU proportion of households with an improved drinking water source within a 30-minute return journey of the household (%)			Per-EU proportion of households with an improved latrine (%)		
	Minimum	Median	Maximum	Minimum	Median	Maximum	Minimum	Median	Maximum
Arsi	28	63	86	24	37	56	0	4	15
Borena	24	66	84	16	24	42	3	9	21
Buno Bedele	62	74	85	52	62	80	0	0	1
East Harerge	44	48	72	34	38	57	0	2	9
East Shewa	28	66	83	26	36	56	2	3	5
East Wellega	52	70	85	47	59	79	2	5	7
Finfine Zuriya	68	68	68	46	46	46	0	0	0
Guji	34	57	76	43	53	63	1	2	5
Illu Aba bora	62	82	100	56	72	76	0	0	2
Jimma	54	73	86	28	36	71	1	3	10
Kelm Wellega	89	91	98	66	79	81	4	7	7
North Shoa	45	72	96	34	50	64	0	2	6
South West Shewa	42	70	95	19	52	74	0	1	4
West Shewa	25	51	84	33	54	71	1	3	13
West Arsi	43	48	96	11	29	60	0	1	7
West Wellega	85	90	95	81	86	87	2	5	6
Total	24	67	100	11	50	87	0	2	21

## Discussion

The baseline trachoma surveys conducted in Oromia region in 2012–2014 as part of the GTMP showed that the TF prevalence in 1–9-year-old children was ≥10% in EUs covering 218 (87%) of the region’s 252 districts, re-establishing the fact that trachoma was a public health problem for a large proportion of the population.^7^ In this set of impact surveys conducted after the requisite number of antibiotic MDA rounds, we demonstrated that the TF prevalence in 1–9-year-olds is now below the 5% elimination target in 73 (56%) of 131 EUs, suggesting that implementation of the A, F and E components of SAFE strategy has been associated with a beneficial impact on reduction of active trachoma prevalence. In line with international guidelines, programs in these districts should now cease antibiotic MDA for 2 years and then re-survey to determine whether the sub-threshold TF prevalence has been sustained.^23^ During that time, implementation of the F and E components of the SAFE strategy should continue in order to limit transmission of ocular *C. trachomatis*.^29^

Elsewhere, post-MDA TF prevalences remained above threshold. Thirty-eight (29%) of the EUs had a TF prevalence between 5.0 and 9.9%, indicating the need for at least one additional round of antibiotic MDA, plus implementation of F and E, before another impact survey. Nineteen (15%) of the EUs had a TF prevalence 10.0–29.9%, for which three rounds of annual MDA and the continued implementation of F and E are required. One district had a TF prevalence of 37.5% and warrants five more rounds of MDA plus F and E implementation. This district in the far north of the region borders other high-prevalence districts in North Shoa zone of Amhara.^30^ Our surveys further showed that in 96 (73%) of surveyed EUs, the age- and gender-adjusted prevalence of TT unknown to the health system was above the 0.2% elimination threshold, indicating outreach and/or static TT surgical intervention, including active case finding, is required in the great majority of districts. Access to improved water sources was particularly heterogeneous across the surveyed EUs and access to an improved latrine was low throughout. There is still some way to go before meeting the sustainable development goal of universal improved water and sanitation facility coverage.^31^ Cross-sectoral working should contribute towards attainment of this universal goal.

**Figure 1.**
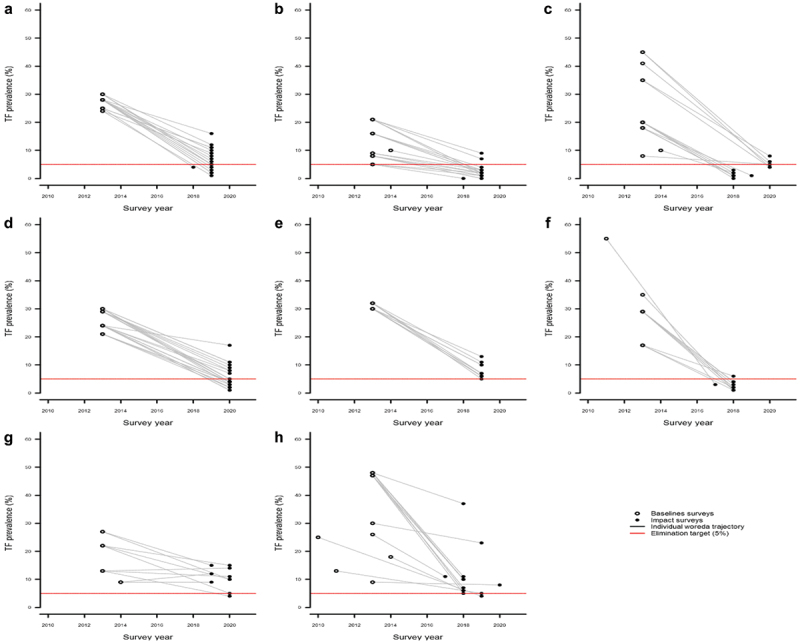
Prevalence of trachomatous inflammation—follicular (TF) among 1–9-year-old children at baseline surveys (2010) and at impact surveys (2017–2020), Oromia Region, Ethiopia.

The large proportion of EUs not achieving elimination prevalence targets despite following international guidelines on SAFE delivery is worrying for the Oromia trachoma elimination programme, but not unique. Other programs in Ethiopia and other countries have also found the recommended number of rounds of MDA insufficient to reach elimination.^32–36^ A number of reasons are behind this which could be further investigated. First, operational parameters including MDA coverage and service quality should be optimised to ensure MDA is being delivered effectively, F and E interventions should be monitored and evaluated to ensure they are making an impact on the hygiene and sanitation behaviours of communities. Second, clinical trials to assess novel strategies for intensified delivery of the SAFE strategy could be carried out. Research into the biology of trachoma in the region could be further investigated: routes of transmission of ocular *C. trachomatis* are only now being more fully explored,^2,4^ while work on the relationship between the background conjunctival microbiome, pathogen genotype, host genotype and susceptibility to infection in different settings is in its infancy.^37–40^All of these factors could potentially contribute to persistence of disease after treatment and warrant further investigation in light of the large number of districts in Oromia where TF has not fallen below 5% after the recommended number of MDA rounds. Both the changing definitions of TT and the different EU framings in place across the lifetime of this programme hinder the direct comparison of TT between pre- and post-GSM4 surveys within this series and limit the comparability of baseline survey data to data from the present surveys. It is clear that the general trajectory of TF prevalence in 1–9-year-old children has been downwards (Figure 1), suggesting that interventions are associated with desirable outcomes. However, direct comparisons beyond that are not possible.

Each point represents a survey in an individual evaluation unit (EU). EUs from neighbouring zones have been grouped together in panels for visualisation purposes. (**a**) Borena, East Harerge and Guji; (**b**) Buno Bedele, Illu Aba Bora, Kelm Wellega and West Wellega; (**c**) East Wellega and Jimma; (**d**) Arsi; (**e**) South West Shewa; (**f**) West Wellega; (**g**) West Arsi; (**h**) East Shewa, Finfine Zuriya and North Shoa. The EUs for pre-mass drug administration (MDA) surveys were formed of several districts (parent EUs), whereas the EUs for post-MDA surveys were mostly formed of single districts (offspring EU); therefore, there are multiple offspring EUs for each parent EU. We caution that the pre-MDA TF prevalence within any parent EU was unlikely to have been sufficiently homogeneous to allow interpolation of pre-MDA TF prevalences for the areas that subsequently became defined as offspring EUs.

## Conclusions

Interventions for trachoma elimination have reduced the prevalence of trachoma in Oromia, but nearly half of the districts still require antibiotic MDA, facial cleanliness and environmental improvement to reach elimination targets, and more than two-thirds require public health-level TT surgery services, including active case finding. Furthermore, substantial efforts to increase access to safe WASH services are required.

## Supplementary Material

Supplemental Material
