# Supplementary material for: Prevalence of Trachoma after Implementation of Trachoma Elimination Interventions in Oromia Regional State, Ethiopia: Results of Impact Surveys in 131 Evaluation Units Covering 139 Districts
Source: Ophthalmic Epidemiol. 2022 Dec 15;30(6):647–54. doi: 10.1080/09286586.2022.2119257 (PMC10581666; doi:10.1080/09286586.2022.2119257)
Supplement: Supplemental Material [file IOPE_A_2119257_SM2784.zip › nope-2021-0327-File002 (1).docx]

**Prevalence of trachoma after implementation of trachoma elimination interventionsin Oromia Regional State, Ethiopia: results of impact surveys in 131 evaluation units covering 139 districts**

**(Supplementary material)**

Annexe:

**Supplementary Table 1**.Population aged ≥1 year participating in trachoma impact surveys in Oromia Region, Ethiopia, February 2017–March 2020.

| Zone | District(s) | Evaluation Unit ID | Date completed (month-year) | Enumerated  (n) | Absent  (n) | Refused  (n) | Examined (n) | Total female examined (n) | Examined female (%) |
| --- | --- | --- | --- | --- | --- | --- | --- | --- | --- |
| Arsi | Amigna | 81146 | 03-2020 | 3003 | 75 | 5 | 2923 | 1614 | 55 |
| Arsi | Aseko | 81147 | 02-2020 | 2865 | 126 | 3 | 2736 | 1476 | 54 |
| Arsi | Bale | 81148 | 03-2020 | 3098 | 118 | 2 | 2978 | 1600 | 54 |
| Arsi | Chole | 81149 | 02-2020 | 2874 | 76 | 21 | 2777 | 1543 | 56 |
| Arsi | Digelu & Tijo | 81150 | 01-2020 | 2994 | 174 | 6 | 2814 | 1540 | 55 |
| Arsi | Diksis | 81151 | 02-2020 | 2906 | 40 | 10 | 2856 | 1625 | 57 |
| Arsi | Dodota | 81152 | 02-2020 | 2928 | 125 | 3 | 2800 | 1478 | 53 |
| Arsi | Enkelo Wabe | 81153 | 01-2020 | 2904 | 139 | 4 | 2761 | 1499 | 54 |
| Arsi | Gololcha | 81154 | 02-2020 | 2948 | 76 | 22 | 2850 | 1551 | 54 |
| Arsi | Guna | 81155 | 02-2020 | 2906 | 176 | 2 | 2728 | 1502 | 55 |
| Arsi | Hetosa | 81156 | 02-2020 | 3021 | 166 | 1 | 2854 | 1520 | 53 |
| Arsi | Jeju | 81157 | 02-2020 | 3142 | 173 | 2 | 2967 | 1606 | 54 |
| Arsi | Limuna bilbilo | 81158 | 01-2020 | 2982 | 125 | 4 | 2852 | 1556 | 55 |
| Arsi | Lode hetosa | 81159 | 02-2020 | 2681 | 43 | 12 | 2626 | 1431 | 54 |
| Arsi | Merti | 81160 | 02-2020 | 2768 | 62 | 17 | 2689 | 1491 | 55 |
| Arsi | Munesa | 81161 | 01-2020 | 3100 | 113 | 3 | 2984 | 1614 | 54 |
| Arsi | Robe | 81162 | 03-2020 | 3164 | 193 | 3 | 2968 | 1625 | 55 |
| Arsi | Seru | 81163 | 03-2020 | 2981 | 105 | 5 | 2871 | 1541 | 54 |
| Arsi | Shanan Kolu | 81164 | 02-2020 | 3064 | 34 | 12 | 3018 | 1690 | 56 |
| Arsi | Shirka | 81165 | 01-2020 | 3127 | 182 | 4 | 2941 | 1589 | 54 |
| Arsi | Sire | 81166 | 02-2020 | 3044 | 124 | 4 | 2916 | 1537 | 53 |
| Arsi | Sude | 81167 | 03-2020 | 3062 | 94 | 4 | 2964 | 1594 | 54 |
| Arsi | Tena | 81168 | 03-2020 | 2874 | 168 | 4 | 2702 | 1517 | 56 |
| Arsi | Tiyo | 81169 | 02-2020 | 3004 | 134 | 10 | 2860 | 1543 | 54 |
| Arsi | Zuway dugda | 81170 | 02-2020 | 3229 | 157 | 7 | 3065 | 1635 | 53 |
| Borena | Dhas | 81053 | 6-2019 | 2671 | 68 | 1 | 2602 | 1567 | 60 |
| Borena | Dire | 81055 | 6-2019 | 2787 | 101 | 4 | 2682 | 1568 | 58 |
| Borena | Dubluk | 81056 | 6-2019 | 2842 | 105 | 8 | 2729 | 1666 | 61 |
| Borena | Eliwaye | 81058 | 6-2019 | 2821 | 114 | 6 | 2701 | 1523 | 56 |
| Borena | Gomole | 81059 | 6-2019 | 3147 | 123 | 11 | 3013 | 1654 | 55 |
| Borena | Guchi | 81062 | 7-2019 | 2539 | 75 | 5 | 2459 | 1509 | 61 |
| Borena | Miyo | 81057 | 7-2019 | 2804 | 99 | 9 | 2696 | 1601 | 59 |
| Borena | Moyale | 81063 | 7-2019 | 2810 | 103 | 5 | 2702 | 1617 | 60 |
| Borena | Teltele | 81060 | 7-2019 | 2962 | 137 | 4 | 2821 | 1525 | 54 |
| Borena | Wachile | 81054 | 7-2019 | 2681 | 64 | 1 | 2616 | 1557 | 60 |
| Borena | Yabelo | 81061 | 6-2019 | 2993 | 118 | 7 | 2868 | 1645 | 57 |
| Buno Bedele | Bedele Rural | 80799 | 4-2019 | 3281 | 226 | 13 | 3042 | 1591 | 52 |
| Buno Bedele | Borecha | 80800 | 4-2019 | 3538 | 261 | 6 | 3270 | 1698 | 52 |
| Buno Bedele | Chora | 80802 | 3-2019 | 3495 | 260 | 5 | 3230 | 1771 | 55 |
| Buno Bedele | Dabo Hana & Chewaka | 80801 | 4-2019 | 3477 | 228 | 8 | 3241 | 1688 | 52 |
| Buno Bedele | Diediesa | 80804 | 4-2019 | 3392 | 270 | 8 | 3114 | 1673 | 54 |
| Buno Bedele | Meko & Dega | 80803 | 3-2019 | 3302 | 298 | 7 | 2997 | 1634 | 55 |
| E. Harerge | Haramaya | 80805 | 4-2019 | 3119 | 213 | 12 | 2894 | 1640 | 57 |
| E. Harerge | Kombolcha | 80543 | 1-2018 | 3475 | 190 | 3 | 3282 | 1720 | 52 |
| E. Harerge | Kurfacale | 80806 | 4-2019 | 3130 | 181 | 6 | 2943 | 1573 | 53 |
| E. Shewa | Adama | 81236 | 2-2020 | 2835 | 128 | 0 | 2707 | 1431 | 53 |
| E. Shewa | Adami Tulu | 80545 | 12-2017 | 3179 | 253 | 5 | 2921 | 1563 | 54 |
| E. Shewa | Fentale | 80809 | 4-2019 | 3005 | 168 | 8 | 2829 | 1510 | 53 |
| E. Shewa | Lome | 80810 | 4-2019 | 3453 | 280 | 21 | 3152 | 1625 | 52 |
| E. Wellega | Diga | 80812 | 12-2018 | 3394 | 177 | 0 | 3217 | 1718 | 53 |
| E. Wellega | Gudaya Bila & Gubu Sayo | 80813 | 11-2018 | 3383 | 180 | 2 | 3200 | 1676 | 52 |
| E. Wellega | Guto Gida | 80814 | 12-2018 | 3457 | 247 | 0 | 3210 | 1719 | 54 |
| E. Wellega | Jima Arjo | 80815 | 12-2018 | 3510 | 197 | 0 | 3313 | 1741 | 53 |
| E. Wellega | Leka Dulecha | 80816 | 12-2018 | 3572 | 189 | 1 | 3382 | 1810 | 54 |
| E. Wellega | Nunu Kumba | 80817 | 12-2018 | 3632 | 196 | 3 | 3433 | 1890 | 55 |
| E. Wellega | Sasiga | 80818 | 5-2019 | 3401 | 179 | 3 | 3219 | 1725 | 54 |
| E. Wellega | Sibu Sire | 80819 | 11-2018 | 3334 | 227 | 1 | 3106 | 1638 | 53 |
| E. Wellega | W/Hagalo & Boneya Bushe | 80811 | 11-2018 | 3601 | 153 | 5 | 3443 | 1765 | 51 |
| E. Wellega | Wayu Tuka | 80820 | 11-2018 | 3373 | 213 | 1 | 3159 | 1695 | 54 |
| Finfine Zuria | Akaki | 80821 | 4-2019 | 3334 | 306 | 22 | 3006 | 1556 | 52 |
| Guji | Anna Soraa | 81078 | 7-2019 | 3247 | 60 | 8 | 3179 | 1705 | 54 |
| Guji | Bore | 81084 | 7-2019 | 3272 | 83 | 2 | 3187 | 1739 | 55 |
| Guji | Dama | 81079 | 7-2019 | 3229 | 73 | 3 | 3153 | 1701 | 54 |
| Guji | Haro Wolabu | 81085 | 7-2019 | 3139 | 69 | 1 | 3069 | 1707 | 56 |
| Guji | Uraga | 81086 | 7-2019 | 3108 | 81 | 0 | 3027 | 1683 | 56 |
| Illu Aba bora | Alge Sachi | 80822 | 3-2019 | 3626 | 272 | 2 | 3352 | 1764 | 53 |
| Illu Aba bora | Bilo Nopa | 80824 | 3-2019 | 3818 | 299 | 7 | 3511 | 1830 | 52 |
| Illu Aba bora | Bure | 80825 | 3-2019 | 3260 | 234 | 3 | 3023 | 1600 | 53 |
| Illu Aba bora | Darimu | 80826 | 3-2019 | 3498 | 273 | 7 | 3218 | 1718 | 53 |
| Illu Aba bora | Didu | 80827 | 3-2019 | 3222 | 222 | 10 | 2990 | 1606 | 54 |
| Illu Aba bora | Doreni | 80828 | 3-2019 | 3339 | 291 | 5 | 3043 | 1596 | 52 |
| Illu Aba bora | Hurumu & Becho | 80823 | 3-2019 | 3261 | 118 | 12 | 3131 | 1626 | 52 |
| Illu Aba bora | Metu | 80546 | 1-2018 | 3472 | 360 | 4 | 3108 | 1634 | 53 |
| Illu Aba bora | Yayo | 80829 | 3-2019 | 3299 | 294 | 7 | 2998 | 1584 | 53 |
| Jimma | Chora & Boter Tolay | 80547 | 1-2018 | 3034 | 216 | 1 | 2817 | 1526 | 54 |
| Jimma | Deddo | 81192 | 03-2020 | 3074 | 88 | 2 | 2984 | 1568 | 53 |
| Jimma | Kersa | 81196 | 03-2020 | 3018 | 80 | 5 | 2933 | 1602 | 55 |
| Jimma | Limmu Kossa | 80553 | 01-2018 | 3610 | 316 | 3 | 3291 | 1719 | 52 |
| Jimma | Limmu Seka | 80548 | 01-2018 | 2931 | 232 | 5 | 2694 | 1433 | 53 |
| Jimma | Mancho | 81197 | 03-2020 | 3034 | 73 | 1 | 2960 | 1702 | 58 |
| Jimma | Shabe Sombo | 81202 | 03-2020 | 2989 | 122 | 5 | 2862 | 1534 | 54 |
| Jimma | Sokoru | 81204 | 03-2020 | 3140 | 127 | 6 | 3007 | 1681 | 56 |
| Jimma | Tiro Afeta | 81205 | 03-2020 | 3078 | 93 | 3 | 2982 | 1608 | 54 |
| K. Wellega | Dale sedi | 80831 | 6-2019 | 3390 | 165 | 2 | 3223 | 1690 | 52 |
| K. Wellega | Lalo Kile | 80903 | 6-2019 | 3364 | 202 | 3 | 3159 | 1697 | 54 |
| Kelem Wellega | Seden Chanka & Dal Wabera | 80832 | 6-2019 | 3296 | 173 | 2 | 3121 | 1751 | 56 |
| North Shoa | Debre Libanos | 80835 | 12-2018 | 3437 | 334 | 20 | 3081 | 1622 | 53 |
| North Shoa | Degem | 80836 | 12-2018 | 3363 | 314 | 27 | 3022 | 1597 | 53 |
| North Shoa | Derra | 80837 | 4-2019 | 3110 | 157 | 12 | 2941 | 1584 | 54 |
| North Shoa | Girar Jarso | 80838 | 12-2018 | 3369 | 252 | 14 | 3103 | 1591 | 51 |
| North Shoa | Jidda & Abichugna | 80552 | 12-2017 | 3039 | 310 | 12 | 2717 | 1431 | 53 |
| North Shoa | Kuyu | 80839 | 12-2018 | 3400 | 189 | 10 | 3201 | 1655 | 52 |
| North Shoa | Were Jarso | 80840 | 12-2018 | 3385 | 321 | 11 | 3053 | 1681 | 55 |
| North Shoa | Wuchale | 80841 | 12-2018 | 3520 | 379 | 8 | 3133 | 1653 | 53 |
| North Shoa | Yaya Gulale | 80842 | 12-2018 | 3282 | 259 | 27 | 2995 | 1546 | 52 |
| S.W. Shewa | Ameya | 81206 | 12-2019 | 3132 | 169 | 11 | 2952 | 1615 | 55 |
| S.W. Shewa | Becho | 81207 | 12-2019 | 3155 | 152 | 8 | 2995 | 1654 | 55 |
| S.W. Shewa | Dawo | 81208 | 12-2019 | 3268 | 129 | 10 | 3129 | 1683 | 54 |
| S.W. Shewa | Elu | 81209 | 12-2019 | 2964 | 119 | 15 | 2830 | 1667 | 59 |
| S.W. Shewa | Goro | 81210 | 12-2019 | 3100 | 165 | 2 | 2933 | 1585 | 54 |
| S.W. Shewa | Kersa Malima | 81211 | 12-2019 | 2994 | 155 | 9 | 2830 | 1538 | 54 |
| S.W. Shewa | Seden Sodo R | 81212 | 12-2019 | 3039 | 123 | 8 | 2908 | 1589 | 55 |
| S.W. Shewa | Sodo Dachi | 81213 | 12-2019 | 2991 | 166 | 13 | 2812 | 1533 | 55 |
| S.W. Shewa | Tole | 81214 | 12-2019 | 3043 | 123 | 11 | 2909 | 1657 | 57 |
| S.W. Shewa | Woliso | 81215 | 12-2019 | 2943 | 159 | 4 | 2780 | 1518 | 55 |
| S.W. Shewa | Wonchi | 81216 | 12-2019 | 3106 | 181 | 8 | 2917 | 1536 | 53 |
| W. Shewa | Ambo Zuria | 80843 | 11-2018 | 3551 | 204 | 5 | 3342 | 1674 | 50 |
| W. Shewa | Chobi | 80844 | 10-2018 | 3543 | 202 | 10 | 3331 | 1756 | 53 |
| W. Shewa | Dendi | 80845 | 10-2018 | 3248 | 246 | 12 | 2990 | 1530 | 51 |
| W. Shewa | Dire Inchini | 80846 | 11-2018 | 3515 | 174 | 5 | 3336 | 1718 | 51 |
| W. Shewa | Ejersa Lafo | 80847 | 10-2018 | 3534 | 459 | 30 | 3045 | 1663 | 55 |
| W. Shewa | Elfeta | 80848 | 11-2018 | 3583 | 160 | 0 | 3423 | 1767 | 52 |
| W. Shewa | Gindeberet | 80849 | 12-2018 | 3818 | 296 | 3 | 3519 | 1876 | 53 |
| W. Shewa | Jeldu | 80850 | 10-2018 | 3387 | 179 | 6 | 3202 | 1725 | 54 |
| W. Shewa | Jibat | 80851 | 11-2018 | 3568 | 132 | 1 | 3435 | 1784 | 52 |
| W. Shewa | Liben Jawi | 80852 | 11-2018 | 3555 | 155 | 1 | 3399 | 1836 | 54 |
| W. Shewa | Nono | 80853 | 11-2018 | 3399 | 114 | 2 | 3283 | 1724 | 53 |
| W. Shewa | Toke Kutaye | 80854 | 11-2018 | 3442 | 186 | 3 | 3253 | 1694 | 52 |
| West Arsi | Adaba | 81217 | 01-2020 | 2943 | 156 | 3 | 2784 | 1566 | 56 |
| West Arsi | Arsi Negele | 80855 | 5-2019 | 3313 | 338 | 11 | 2964 | 1625 | 55 |
| West Arsi | Dodola | 81218 | 01-2020 | 3285 | 148 | 10 | 3127 | 1737 | 56 |
| West Arsi | Gadeb Asasa | 81219 | 01-2020 | 3220 | 141 | 11 | 3068 | 1769 | 58 |
| West Arsi | Heben Arsi | 80856 | 06-2019 | 2871 | 187 | 5 | 2679 | 1502 | 56 |
| West Arsi | Kofele | 81220 | 01-2020 | 3270 | 168 | 3 | 3099 | 1764 | 57 |
| West Arsi | Kokosa | 81221 | 01-2020 | 3067 | 116 | 0 | 2951 | 1708 | 58 |
| West Arsi | Kore | 81222 | 01-2020 | 3381 | 144 | 3 | 3234 | 1820 | 56 |
| West Arsi | Nensebo | 81223 | 01-2020 | 3074 | 112 | 5 | 2957 | 1656 | 56 |
| West Arsi | Wondo | 81224 | 12-2019 | 3229 | 136 | 1 | 3092 | 1671 | 54 |
| W. Wellega | Begi | 80857 | 5-2019 | 3559 | 228 | 1 | 3330 | 1799 | 54 |
| W. Wellega | Kiltu Kara | 80859 | 5-2019 | 3380 | 151 | 2 | 3227 | 1692 | 52 |
| W. Wellega | Yubdo&homa | 80858 | 5-2019 | 3309 | 173 | 2 | 3134 | 1682 | 54 |
| Total | |  |  | 418,830 | 22,802 | 855 | 395,166 | 214,076 | 54 |

**Supplementary Table 2**. Cases of active (inflammatory) trachoma and age-adjusted prevalence of trachomatous inflammation—follicular(TF) among 1–9-year-old children in 131 trachoma impact surveys, Oromia Region, Ethiopia, February 2017–March 2020.

| Zone | District(s) | Children aged 1−9 years examined (n) | Children aged 1−9 years with TF in one or both eyes (n) | Children aged 1−9 years with TI in one or both eyes (n) | Age-adjusted prevalence of TF in 1−9-year-olds (%, 95% CI) |
| --- | --- | --- | --- | --- | --- |
| Arsi | Amigna | 1,156 | 140 | 8 | 11.37 (7.95−15.57) |
| Arsi | Aseko | 952 | 38 | 1 | 3.60 (1.64−6.26) |
| Arsi | Bale | 1,044 | 48 | 1 | 3.97 (2.48−5.43) |
| Arsi | Chole | 799 | 10 | 1 | 0.80 (0.17−1.79) |
| Arsi | Digelu & Tijo | 1,013 | 40 | 3 | 3.49 (1.94−5.36) |
| Arsi | Diksis | 1,014 | 97 | 4 | 7.68 (4.78−11.48) |
| Arsi | Dodota | 957 | 87 | 9 | 10.03 (6.25−15.21) |
| Arsi | Enkelo Wabe | 953 | 19 | 0 | 1.64 (0.61−2.69) |
| Arsi | Gololcha | 994 | 52 | 10 | 4.42 (2.47−6.50) |
| Arsi | Guna | 970 | 7 | 1 | 0.62 (0.11−1.34) |
| Arsi | Hetosa | 941 | 36 | 5 | 3.79 (1.69−6.62) |
| Arsi | Jeju | 1,141 | 56 | 2 | 5.07 (2.91−8.04) |
| Arsi | Limuna bilbilo | 1,066 | 20 | 1 | 2.01 (0.95−3.10) |
| Arsi | Lode hetosa | 838 | 48 | 3 | 4.05 (2.50−5.72) |
| Arsi | Merti | 955 | 98 | 18 | 7.83 (4.38−11.64) |
| Arsi | Munesa | 1,132 | 38 | 1 | 3.51 (1.90−5.74) |
| Arsi | Robe | 1,065 | 55 | 3 | 4.51 (2.71−6.46) |
| Arsi | Seru | 1,227 | 163 | 4 | 11.21 (8.06−15.56) |
| Arsi | Shanan Kolu | 1,248 | 109 | 14 | 7.33 (4.81−10.46) |
| Arsi | Shirka | 1,047 | 35 | 2 | 2.94 (1.44−5.13) |
| Arsi | Sire | 940 | 51 | 5 | 4.38 (2.72−6.58) |
| Arsi | Sude | 1,161 | 113 | 8 | 8.58 (5.76−12.02) |
| Arsi | Tena | 869 | 35 | 2 | 3.70 (1.93−6.11) |
| Arsi | Tiyo | 957 | 23 | 3 | 2.01 (0.74−3.67) |
| Arsi | Zuway dugda | 1,202 | 211 | 16 | 16.69 (12.46−20.14) |
| Borena | Dhas | 1,186 | 209 | 9 | 15.78 (12.02−19.88) |
| Borena | Wachile | 1,150 | 118 | 2 | 8.09 (5.97−10.33) |
| Borena | Dire | 1,088 | 51 | 3 | 4.09(2.57−5.90) |
| Borena | Dubluk | 1,118 | 36 | 2 | 2.85(1.61−4.38) |
| Borena | Miyo | 1,115 | 55 | 2 | 4.57 (2.67−7.09) |
| Borena | Eliwaye | 1,140 | 29 | 0 | 1.88 (0.93−3.07) |
| Borena | Gomole | 1,338 | 75 | 4 | 5.01 (3.01−7.62) |
| Borena | Teltele | 1,188 | 76 | 17 | 6.85 (4.17−10.25) |
| Borena | Yabelo | 1,204 | 57 | 2 | 4.68 (2.95−6.82) |
| Borena | Guchi | 1,215 | 127 | 1 | 9.55 (6.78−12.88) |
| Borena | Moyale | 1,212 | 29 | 4 | 2.00 (1.02−3.04) |
| Buno Bedele | Bedele Rural | 1,041 | 17 | 8 | 1.51 (0.58−2.84) |
| Buno Bedele | Borecha | 1,273 | 93 | 26 | 7.29 (4.62−10.73) |
| Buno Bedele | DaboHana & Chewaka | 1,292 | 49 | 4 | 3.36 (1.76−5.41) |
| Buno Bedele | Chora | 1,050 | 27 | 5 | 2.59 (1.10−3.98) |
| Buno Bedele | Meko & Dega | 991 | 16 | 1 | 1.91 (0.92−3.14) |
| Buno Bedele | Diediesa | 1,103 | 104 | 20 | 9.31 (6.84−12.62) |
| E. Harerge | Kombolcha | 1,215 | 51 | 0 | 4.18 (2.68−6.15) |
| E. Harerge | Haramaya | 1,193 | 139 | 12 | 11.15 (7.61−15.83) |
| E. Harerge | Kurfacale | 1,123 | 127 | 13 | 11.04 (8.42−13.61) |
| E. Shewa | Adami Tulu | 1,102 | 63 | 10 | 6.05 (3.90−8.67) |
| E. Shewa | Fentale | 1,160 | 56 | 6 | 4.22 (2.47−6.43) |
| E. Shewa | Lome | 885 | 46 | 3 | 4.96 (2.96−6.91) |
| E.Shewa | Adama | 873 | 82 | 7 | 8.19 (4.99−12.57) |
| E. Wellega | Wama Hagalo & Boneya Bushe | 1,274 | 11 | 2 | 0.89 (0.36−1.58) |
| E. Wellega | Diga | 981 | 7 | 2 | 0.57 (0.20−1.06) |
| E. Wellega | Gudaya Bila & Gubu Sayo | 1,086 | 6 | 0 | 0.59 (0.08−1.40) |
| E. Wellega | Guto Gida | 978 | 9 | 0 | 1.00 (0.20−1.64) |
| E. Wellega | Jima Arjo | 1,082 | 4 | 1 | 0.29 (0.06−0.55) |
| E. Wellega | Leka Dulecha | 1,139 | 28 | 4 | 2.58 (1.28−4.33) |
| E. Wellega | Nunu Kumba | 1,248 | 23 | 1 | 1.98 (0.88−3.42) |
| E. Wellega | Sasiga | 1,174 | 13 | 2 | 1.17 (0.30−2.41) |
| E. Wellega | Sibu Sire | 1,140 | 14 | 1 | 1.51 (0.40−3.10) |
| E. Wellega | Wayu Tuka | 1,007 | 11 | 2 | 0.72 (0.26−1.33) |
| Finfine Zuriya | Akaki | 888 | 213 | 20 | 22.73 (17.81−27.03) |
| Guji | Anna Soraa | 1,475 | 134 | 10 | 8.53 (6.35−11.05) |
| Guji | Dama | 1,430 | 15 | 0 | 1.18 (0.45−2.20) |
| Guji | Bore | 1,375 | 72 | 6 | 5.90 (2.54−10.02) |
| Guji | Haro Wolabu | 1,474 | 90 | 9 | 5.19 (3.09−7.87) |
| Guji | Uraga | 1,406 | 179 | 14 | 11.98 (8.97−15.27) |
| Illu Aba bora | Metu | 679 | 2 | 0 | 0.16 (0.00−0.32) |
| Illu Aba bora | Alge Sachi | 886 | 10 | 1 | 1.20 (0.45−2.28) |
| Illu Aba bora | Hurumu & Becho | 1,034 | 20 | 3 | 1.85 (0.81−3.03) |
| Illu Aba bora | Bilo Nopa | 977 | 11 | 0 | 1.65 (0.36−3.63) |
| Illu Aba bora | Bure | 1,068 | 19 | 0 | 2.04 (0.85−3.45) |
| Illu Aba bora | Darimu | 1,072 | 28 | 5 | 2.55 (1.54−3.79) |
| Illu Aba bora | Didu | 1,057 | 34 | 4 | 3.23 (1.77−5.08) |
| Illu Aba bora | Doreni | 894 | 29 | 4 | 3.58 (2.26−5.13) |
| Illu Aba bora | Yayo | 860 | 13 | 3 | 1.41 (0.16−2.76) |
| Jimma | Chora&Boter Tolay | 965 | 19 | 1 | 1.82 (0.44−3.97) |
| Jimma | Limmu Seka | 944 | 25 | 1 | 2.31 (0.64−4.62) |
| Jimma | Limmu Kossa | 965 | 48 | 3 | 4.46 (2.68−6.75) |
| Jimma | Deddo | 1,061 | 46 | 1 | 4.35 (2.41−7.09) |
| Jimma | Kersa | 1,005 | 45 | 1 | 4.45 (1.89−6.34) |
| Jimma | Mancho | 1,178 | 59 | 2 | 5.68 (3.31−9.18) |
| Jimma | Shabe Sombo | 1,069 | 60 | 2 | 5.43 (2.84−8.86) |
| Jimma | Sokoru | 1,151 | 72 | 1 | 5.38 (3.38−7.48) |
| Jimma | Tiro Afeta | 1,113 | 99 | 5 | 8.30 (5.96−11.18) |
| Kelem Wellega | Dale sedi | 898 | 4 | 2 | 0.31 (0.04−0.65) |
| Kelem Wellega | Seden Chanka & Dale Wabera | 877 | 9 | 0 | 0.92 (0.13−2.12) |
| Kelem Wellega | Lalo Kile | 922 | 13 | 1 | 1.29 (0.58−2.24) |
| North Shoa | Jida & Abichugna | 865 | 91 | 10 | 10.73 (7.47−14.18) |
| North Shoa | Debre Libanos | 1,145 | 79 | 9 | 6.86 (4.28−9.92) |
| North Shoa | Degem | 1,092 | 54 | 7 | 5.59 (2.85−9.45) |
| North Shoa | Derra | 870 | 337 | 57 | 37.49 (31.14−43.68) |
| North Shoa | Girar Jarso | 1,084 | 96 | 6 | 9.55 (6.05−14.09) |
| North Shoa | Kuyu | 1,136 | 98 | 13 | 9.54 (6.18−13.59) |
| North Shoa | Were Jarso | 1,054 | 96 | 12 | 10.98 (8.31−14.37) |
| North Shoa | Wuchale | 1,155 | 119 | 21 | 9.93 (7.67−12.24) |
| North Shoa | Yaya Gulale | 1,095 | 53 | 6 | 5.24 (3.55−7.39) |
| S.W. Shewa | Ameya | 1,110 | 66 | 4 | 5.59 (3.66−7.99) |
| S.W. Shewa | Becho | 990 | 66 | 1 | 6.53 (4.42−8.78) |
| S.W. Shewa | Dawo | 1,049 | 100 | 5 | 9.80 (6.92−13.30) |
| S.W. Shewa | Elu | 920 | 55 | 2 | 7.02 (3.79−10.04) |
| S.W. Shewa | Goro | 1,081 | 111 | 8 | 10.76 (6.75−15.36) |
| S.W. Shewa | Kersa Malima | 995 | 68 | 5 | 5.88 (3.59−8.96) |
| S.W. Shewa | Seden Sodo Rural | 1,124 | 107 | 7 | 9.95 (5.72−13.87) |
| S.W. Shewa | Sodo Dachi | 986 | 146 | 16 | 12.77 (7.78−19.08) |
| S.W. Shewa | Tole | 1,036 | 63 | 4 | 5.94 (3.96−8.04) |
| S.W. Shewa | Woliso | 911 | 66 | 3 | 7.29 (3.87−11.03) |
| S.W. Shewa | Wonchi | 954 | 47 | 3 | 5.07 (3.27−7.39) |
| W. Shewa | Ambo Zuria | 1,189 | 28 | 2 | 2.74 (1.43−4.44) |
| W. Shewa | Chobi | 1,180 | 48 | 3 | 4.15 (2.26−6.51) |
| W. Shewa | Dendi | 1,010 | 16 | 4 | 1.50 (0.69−2.56) |
| W. Shewa | Dire Inchini | 1,231 | 16 | 3 | 1.24 (0.52−1.93) |
| W. Shewa | Ejersa Lafo | 888 | 39 | 5 | 3.55 (2.17−5.26) |
| W. Shewa | Elfeta | 1,271 | 11 | 0 | 0.89 (0.37−1.57) |
| W. Shewa | Gindeberet | 1,146 | 62 | 2 | 6.13 (2.93−9.42) |
| W. Shewa | Jeldu | 1,126 | 21 | 3 | 2.26 (1.34−3.48) |
| W. Shewa | Jibat | 1,280 | 12 | 1 | 0.99 (0.40−1.74) |
| W. Shewa | Liben Jawi | 1,298 | 42 | 4 | 3.50 (2.17−4.87) |
| W. Shewa | Nono | 1,212 | 30 | 2 | 2.77 (1.73−4.15) |
| W. Shewa | Toke Kutaye | 1,211 | 22 | 2 | 1.56 (0.59−2.88) |
| West Arsi | Arsi Negele | 1,176 | 157 | 15 | 11.71 (8.48−14.98) |
| West Arsi | Heben Arsi | 1,098 | 96 | 6 | 8.84 (5.83−12.72) |
| West Arsi | Adaba | 1,087 | 144 | 6 | 11.45 (6.73−15.93) |
| West Arsi | Dodola | 1,344 | 145 | 11 | 9.65 (6.82−13.42) |
| West Arsi | Gadeb Asasa | 1,288 | 196 | 6 | 14.66 (10.62−19.51) |
| West Arsi | Kofele | 1,394 | 144 | 5 | 10.44 (6.87−14.78) |
| West Arsi | Kokosa | 1,245 | 55 | 1 | 4.13 (2.14−6.96) |
| West Arsi | Kore | 1,325 | 63 | 3 | 4.91 (3.16−7.13) |
| West Arsi | Nensebo | 1,265 | 161 | 4 | 13.60 (8.39−19.81) |
| West Arsi | Wondo | 1,180 | 174 | 9 | 15.10 (11.31−19.52) |
| West Wellega | Begi | 1,149 | 31 | 3 | 1.97 (0.95−2.97) |
| West Wellega | Yubdo & Homa | 842 | 3 | 0 | 0.15 (0.00−0.36) |
| West Wellega | Kiltu Kara | 850 | 9 | 0 | 1.21 (0.30−2.51) |
| Total | | 142,665 | 8,498 | 699 |  |
| CI: confidence interval; TF: trachomatous inflammation─follicular; TI: trachomatous inflammation─intense. | | | | | |

**Supplementary Table 3.** Age- and gender-adjusted prevalence of trachomatous trichiasis (TT) unknown to the health system in those aged ≥15 years in 51 trachoma impact surveys in Oromia Region, Ethiopia, December 2019- March 2020. In these EUs, the post-GSM4 definition of TT was used, in which only upper-lid involvement constituted TT.

| **Zone** | **District(s)** | **Population aged ≥15 years examined (n)** | **Cases of TT (management status not specified) in population aged ≥15 years (n)** | **Cases of TT unknown to the health system in population aged ≥15 years (n)** | **Adjusted prevalence of TT unknown to the health system in ≥15-year-olds (%, 95% confidence interval)** |
| --- | --- | --- | --- | --- | --- |
| Arsi | Amigna | 1,464 | 13 | 12 | 0.61 (0.24–1.01) |
|  | Aseko | 1,556 | 24 | 19 | 0.54 (0.29–0.83) |
|  | Bale | 1,584 | 13 | 12 | 0.61 (0.25–0.96) |
|  | Chole | 1,691 | 5 | 5 | 0.09 (0.02–0.16) |
|  | Digelu & Tijo | 1,476 | 1 | 1 | 0.03 (0.00–0.09) |
|  | Diksis | 1,473 | 3 | 2 | 0.06 (0.00–0.16) |
|  | Dodota | 1,544 | 34 | 28 | 1.05 (0.58–1.67) |
|  | Enkelo Wabe | 1,494 | 6 | 5 | 0.10 (0.00–0.21) |
|  | Gololcha | 1,455 | 5 | 3 | 0.17 (0.00–0.41) |
|  | Guna | 1,488 | 3 | 2 | 0.14 (0.00–0.38) |
|  | Hetosa | 1,613 | 17 | 15 | 0.50 (0.28–0.80) |
|  | Jeju | 1,500 | 8 | 6 | 0.24 (0.05–0.56) |
|  | Limuna bilbilo | 1,469 | 3 | 3 | 0.10 (0.00–0.25) |
|  | Lode hetosa | 1,522 | 12 | 10 | 0.37 (0.07–0.66) |
|  | Merti | 1,477 | 16 | 11 | 0.39 (0.12–0.77) |
|  | Munesa | 1,492 | 4 | 4 | 0.18 (0.01–0.34) |
|  | Robe | 1,571 | 11 | 8 | 0.41 (0.13–0.80) |
|  | Seru | 1,341 | 27 | 20 | 0.83 (0.45–1.30) |
|  | Shanan Kolu | 1,412 | 14 | 12 | 0.42 (0.22–0.67) |
|  | Shirka | 1,568 | 20 | 17 | 0.61 (0.35–0.90) |
|  | Sire | 1,626 | 19 | 18 | 0.52 (0.19–0.80) |
|  | Sude | 1,481 | 5 | 5 | 0.15 (0.03–0.31) |
|  | Tena | 1,545 | 17 | 12 | 0.58 (0.29–0.96) |
|  | Tiyo | 1,595 | 11 | 5 | 0.21 (0.06–0.40) |
|  | Zuway dugda | 1,514 | 38 | 28 | 1.02 (0.57–1.51) |
| Jimma | Deddo | 1,556 | 29 | 21 | 0.62 (0.31–1.04) |
|  | Kersa | 1,563 | 23 | 14 | 0.78 (0.15–1.78) |
|  | Mancho | 1,432 | 34 | 20 | 0.80 (0.45–1.28) |
|  | Shabe Sombo | 1,478 | 28 | 27 | 1.09 (0.58–1.45) |
|  | Sokoru | 1,511 | 52 | 43 | 2.18 (1.21–3.06) |
|  | Tiro Afeta | 1,549 | 31 | 23 | 0.98 (0.53–1.59) |
| S.W. Shewa | Ameya | 1,441 | 25 | 20 | 0.64 (0.34–1.00) |
|  | Becho | 1,576 | 21 | 19 | 0.99 (0.48–1.56) |
|  | Dawo | 1,630 | 19 | 14 | 0.26 (0.13–0.44) |
|  | Elu | 1,532 | 21 | 20 | 0.59 (0.29–0.96) |
|  | Goro | 1,519 | 58 | 44 | 1.51 (0.80–2.25) |
|  | Kersa Malima | 1,441 | 30 | 27 | 1.07 (0.53–1.84) |
|  | Seden Sodo Rural | 1,398 | 22 | 18 | 0.55 (0.19–0.84) |
|  | Sodo Dachi | 1,459 | 30 | 24 | 0.60 (0.32–0.87) |
|  | Tole | 1,496 | 16 | 16 | 0.44 (0.21–0.66) |
|  | Woliso | 1,514 | 39 | 32 | 1.21 (0.51–2.20) |
|  | Wonchi | 1,584 | 8 | 7 | 0.11 (0.03–0.22) |
| West Arsi | Adaba | 1,368 | 5 | 5 | 0.25 (0.03–0.56) |
|  | Dodola | 1,413 | 6 | 4 | 0.12 (0.03–0.25) |
|  | Gadeb Asasa | 1,400 | 2 | 2 | 0.06 (0.00–0.18) |
|  | Kofele | 1,361 | 2 | 2 | 0.08 (0.00–0.23) |
|  | Kokosa | 1,376 | 2 | 1 | 0.05 (0.00–0.15) |
|  | Kore | 1,498 | 1 | 1 | 0.01 (0.00–0.02) |
|  | Nensebo | 1,385 | 4 | 3 | 0.13 (0.00–0.26) |
|  | Wondo | 1,572 | 25 | 22 | 0.81 (0.35–1.34) |
| E/ Shoa | Adama | 1,562 | 15 | 13 | 0.53 (0.20–0.83 |
| Total | | 76,565 | 877 | 705 |  |

**Supplementary Table 4.**Age- and gender-adjusted prevalence of trachomatous trichiasis (TT) unknown to the health system among adults aged ≥15 years in 80 trachoma impact surveys in Oromia Region, Ethiopia, February 2017–July 2019. In these EUs, the pre-GSM4 definition of TT was used, in which the eyelid of origin of the in-turned or epilated eyelash was not recorded.

| **Zone** | **District(s)** | **Population aged ≥15 years examined (n)** | **Population aged ≥15 years with TT (management status unspecified)(n)** | **Population aged ≥15 years with TT unknown to the health system(n)** | **Age- and gender-adjusted prevalence of TT unknown to the health system in ≥15-year-olds (%, 95% confidence interval)** |
| --- | --- | --- | --- | --- | --- |
| Borena | Dhas | 1223 | 21 | 20 | 0.63 (0.32-0.96) |
| Borena | Wachile | 1249 | 20 | 16 | 0.98 (0.40-1.73) |
| Borena | Dire | 1379 | 22 | 14 | 0.38 (0.13-0.71) |
| Borena | Dubluk | 1338 | 19 | 13 | 0.34 (0.10-0.72) |
| Borena | Miyo | 1347 | 16 | 12 | 0.33 (0.16-0.54) |
| Borena | Eliwaye | 1349 | 15 | 11 | 0.26 (0.08-0.42) |
| Borena | Gomole | 1369 | 11 | 7 | 0.15 (0.05-0.31) |
| Borena | Teltele | 1369 | 15 | 12 | 0.46 (0.23-0.75) |
| Borena | Yabelo | 1390 | 27 | 21 | 0.58 (0.31-0.91) |
| Borena | Guchi | 1085 | 14 | 10 | 0.24 (0.10-0.43) |
| Borena | Moyale | 1257 | 6 | 4 | 0.19 (0.04-0.43) |
| Buno Bedele | Bedele Rural | 1651 | 13 | 12 | 0.55 (0.15-1.11) |
| Buno Bedele | Borecha | 1613 | 25 | 23 | 0.84 (0.46-1.24) |
| Buno Bedele | Dabo Hana&Chewaka | 1568 | 7 | 7 | 0.29 (0.08-0.60) |
| Buno Bedele | Chora | 1733 | 19 | 13 | 0.51 (0.24-0.85) |
| Buno Bedele | Meko & Dega | 1663 | 16 | 13 | 0.41 (0.20-0.68) |
| Buno Bedele | Diediesa | 1632 | 20 | 18 | 0.73 (0.40-1.15) |
| E. Harerge | Kombolcha | 1652 | 14 | 10 | 0.28 (0.10-0.45) |
| E. Harerge | Haramaya | 1400 | 18 | 13 | 0.38 (0.18-0.66) |
| E. Harerge | Kurfacale | 1454 | 27 | 16 | 0.86 (0.35-1.55) |
| E. Shewa | Adami Tulu | 1396 | 16 | 12 | 0.44 (0.23-0.66) |
| E. Shewa | Fentale | 1369 | 9 | 6 | 0.28 (0.05-0.61) |
| E. Shewa | Lome | 1975 | 35 | 23 | 0.77 (0.42-1.13) |
| E. Wellega | Wama Hagalo & Boneya Bushe | 1747 | 10 | 9 | 0.26 (0.09-0.48) |
| E. Wellega | Diga | 1792 | 17 | 11 | 0.31 (0.10-0.51) |
| E. Wellega | Gudaya Bila & Gubu Sayo | 1701 | 17 | 17 | 0.55 (0.25-0.85) |
| E. Wellega | Guto Gida | 1791 | 21 | 21 | 0.74 (0.46-1.09) |
| E. Wellega | Jima Arjo | 1779 | 27 | 20 | 0.98 (0.49-1.63) |
| E. Wellega | Leka Dulecha | 1816 | 58 | 46 | 1.74 (1.13-2.39) |
| E. Wellega | Nunu Kumba | 1716 | 16 | 13 | 0.56 (0.22-1.11) |
| E. Wellega | Sasiga | 1742 | 14 | 11 | 0.41 (0.20-0.71) |
| E. Wellega | Sibu Sire | 1624 | 14 | 9 | 0.30 (0.14-0.49) |
| E. Wellega | Wayu Tuka | 1759 | 34 | 29 | 1.01 (0.52-1.48) |
| Finfine Zuriya | Akaki | 1691 | 33 | 17 | 0.63 (0.23-1.01) |
| Guji | Anna Soraa | 1373 | 3 | 3 | 0.10 (0.00-0.25) |
| Guji | Dama | 1389 | 1 | 1 | 0.04 (0.00-0.11) |
| Guji | Bore | 1462 | 3 | 3 | 0.25 (0.00-0.72) |
| Guji | Haro Wolabu | 1316 | 3 | 3 | 0.08 (0.00-0.21) |
| Guji | Uraga | 1341 | 4 | 2 | 0.05 (0.00-0.15) |
| Illu Aba bora | Metu | 2059 | 2 | 2 | 0.05 (0.00-0.14) |
| Illu Aba bora | Alge Sachi | 2055 | 6 | 5 | 0.14 (0.04-0.27) |
| Illu Aba bora | Hurumu & Becho | 1834 | 5 | 4 | 0.09 (0.00-0.20) |
| Illu Aba bora | Bilo Nopa | 2152 | 6 | 2 | 0.06 (0.00-0.18) |
| Illu Aba bora | Bure | 1706 | 5 | 4 | 0.07 (0.02-0.14) |
| Illu Aba bora | Darimu | 1717 | 7 | 6 | 0.33 (0.08-0.65) |
| Illu Aba bora | Didu | 1657 | 2 | 1 | 0.01 (0.00-0.03) |
| Illu Aba bora | Doreni | 1739 | 16 | 14 | 0.58 (0.23-1.06) |
| Illu Aba bora | Yayo | 1786 | 8 | 6 | 0.20 (0.05-0.39) |
| Jimma | Chora & Boter Tolay | 1516 | 8 | 4 | 0.17 (0.02-0.38) |
| Jimma | Limmu Seka | 1414 | 9 | 5 | 0.18 (0.01-0.33) |
| Jimma | Limmu Kossa | 1913 | 18 | 11 | 0.33 (0.16-0.56) |
| Kelem Wellega | Dale sedi | 1858 | 7 | 4 | 0.15 (0.00-0.35) |
| Kelem Wellega | Seden Chanka & Dale Wabera | 1800 | 12 | 11 | 0.26 (0.10-0.47) |
| Kelem Wellega | Lalo Kile | 1794 | 5 | 4 | 0.35 (0.06-0.75) |
| North Shoa | Jidda & Abichugna | 1516 | 9 | 7 | 0.38 (0.04-0.70) |
| North Shoa | Debre Libanos | 1645 | 21 | 17 | 0.59 (0.25-0.96) |
| North Shoa | Degem | 1657 | 20 | 17 | 0.48 (0.17-0.92) |
| North Shoa | Derra | 1702 | 98 | 64 | 1.87 (1.22-2.61) |
| North Shoa | Girar Jarso | 1734 | 24 | 16 | 0.58 (0.15-1.25) |
| North Shoa | Kuyu | 1702 | 35 | 31 | 1.19 (0.64-1.88) |
| North Shoa | Were Jarso | 1665 | 41 | 25 | 0.57 (0.23-1.03) |
| North Shoa | Wuchale | 1644 | 19 | 15 | 0.31 (0.14-0.54) |
| North Shoa | Yaya Gulale | 1599 | 14 | 10 | 0.38 (0.17-0.67) |
| W. Shewa | Ambo Zuria | 1672 | 15 | 12 | 0.40 (0.16-0.70) |
| W. Shewa | Chobi | 1659 | 19 | 16 | 0.53 (0.25-0.86) |
| W. Shewa | Dendi | 1603 | 5 | 4 | 0.17 (0.00-0.44) |
| W. Shewa | Dire Inchini | 1705 | 6 | 3 | 0.15 (0.00-0.35) |
| W. Shewa | Ejersa Lafo | 1737 | 21 | 20 | 0.61 (0.23-1.23) |
| W. Shewa | Elfeta | 1719 | 4 | 2 | 0.07 (0.00-0.22) |
| W. Shewa | Gindeberet | 1879 | 38 | 21 | 0.68 (0.30-1.04) |
| W. Shewa | Jeldu | 1653 | 10 | 7 | 0.30 (0.08-0.58) |
| W. Shewa | Jibat | 1702 | 12 | 10 | 0.29 (0.11-0.55) |
| W. Shewa | Liben Jawi | 1672 | 15 | 14 | 0.54 (0.22-0.95) |
| W. Shewa | Nono | 1676 | 22 | 18 | 0.89 (0.41-1.49) |
| W. Shewa | Toke Kutaye | 1641 | 7 | 5 | 0.18 (0.03-0.37) |
| West Arsi | Arsi Negele | 1471 | 32 | 23 | 1.14 (0.59-1.85) |
| West Arsi | Heben Arsi | 1341 | 23 | 15 | 0.73 (0.36-1.15) |
| West Wellega | Begi | 1745 | 22 | 18 | 0.58 (0.28-0.87) |
| West Wellega | Yubdo & Homa | 1917 | 3 | 3 | 0.09 (0.00-0.22) |
| West Wellega | Kiltu Kara | 1997 | 7 | 6 | 0.16 (0.04-0.33) |
| Total | | 130,153 | 1338 | 1,003 |  |

**Supplementary Table 5**. Water and sanitation facilities in households surveyed during 131 trachoma impact surveys in Oromia Region, Ethiopia, February 2017−March 2020. Improvement status was defined according to United Nations Children’s Fund/World Health Organization Joint Monitoring Programme definitions.

| Zone | District(s) | Households surveyed (n) | Households with improved drinking water source (n, %) | Households with drinking water source within 30 minutes (n, %) | Households with improved latrine (n, %) |
| --- | --- | --- | --- | --- | --- |
| Arsi | Amigna | 780 | 446 (57) | 220 (28) | 15 (2) |
| Arsi | Aseko | 781 | 219 (28) | 268 (34) | 0 (0) |
| Arsi | Bale | 783 | 507 (65) | 302 (39) | 41 (5) |
| Arsi | Chole | 901 | 467 (52) | 463 (51) | 75 (8) |
| Arsi | Digelu & Tijo | 783 | 432 (55) | 415 (53) | 23 (3) |
| Arsi | Diksis | 780 | 516 66) | 203 (26) | 52 (7) |
| Arsi | Dodota | 784 | 528 (67) | 237 (30) | 35 (4) |
| Arsi | Enkelo Wabe | 783 | 517 (66) | 335 (43) | 14 (2) |
| Arsi | Gololcha | 784 | 292 (37) | 279 (36) | 86 (11) |
| Arsi | Guna | 783 | 466 (60) | 297 (38) | 19 (2) |
| Arsi | Hetosa | 782 | 670 (86) | 427 (55) | 13 (2) |
| Arsi | Jeju | 781 | 512 (66) | 366 (47) | 53 (7) |
| Arsi | Limuna bilbilo | 786 | 320 (41) | 267 (34) | 8 (1) |
| Arsi | Lode hetosa | 780 | 521 (67) | 309 (40) | 116 (15) |
| Arsi | Merti | 779 | 420 (54) | 245 (31) | 87 (11) |
| Arsi | Munesa | 784 | 599 (76) | 293 (37) | 13 (2) |
| Arsi | Robe | 779 | 577 (74) | 315 (40) | 56 (7) |
| Arsi | Seru | 778 | 248 (32) | 284 (37) | 33 (4) |
| Arsi | Shanan Kolu | 778 | 341 (44) | 190 (24) | 23 (3) |
| Arsi | Shirka | 783 | 495 (63) | 440 (56) | 40 (5) |
| Arsi | Sire | 783 | 550 (70) | 237 (30) | 0 (0) |
| Arsi | Sude | 778 | 260 (33) | 247 (32) | 0 (0) |
| Arsi | Tena | 782 | 581 (74) | 378 (48) | 62 (8) |
| Arsi | Tiyo | 787 | 406 (52) | 409 (52) | 59 (7) |
| Arsi | Zuway dugda | 780 | 631 (81) | 287 (37 | 18 (2) |
| Borena | Dhas | 787 | 320 (41) | 125 (16) | 33 (4) |
| Borena | Wachile | 787 | 558 (71) | 239 (30) | 135 (17) |
| Borena | Dire | 784 | 515 (66) | 159 (20) | 100 (13) |
| Borena | Dubluk | 782 | 523 (67) | 230 (29) | 86 (11) |
| Borena | Miyo | 787 | 543 (69) | 188 (24) | 47 (6) |
| Borena | Eliwaye | 784 | 199 (25) | 192 (24) | 62 (8) |
| Borena | Gomole | 783 | 186 (24) | 168 (21) | 20 (3) |
| Borena | Teltele | 790 | 230 (29) | 220 (28) | 50 (6) |
| Borena | Yabelo | 786 | 380 (48) | 162 (21) | 68 (9) |
| Borena | Guchi | 783 | 660 (84) | 189 (24) | 103 (13) |
| Borena | Moyale | 786 | 613 (78) | 330 (42) | 164 (21) |
| Buno Bedele | Bedele Rural | 801 | 576 (72) | 506 (63) | 6 (1) |
| Buno Bedele | Borecha | 784 | 667 (85) | 404 (52) | 1 (<1) |
| Buno Bedele | Dabo Hana&Chewaka | 783 | 488 (62) | 433 (55) | 1 (<1) |
| Buno Bedele | Chora | 806 | 540 (67) | 543 (67) | 1 (<1) |
| Buno Bedele | Meko & Dega | 810 | 624 (77) | 645 (80) | 1 (<1) |
| Buno Bedele | Diediesa | 791 | 613 (77) | 474 (60) | 1 (<1) |
| E. Harerge | Kombolcha | 774 | 554 (72) | 442 (57) | 68 (9) |
| E. Harerge | Haramaya | 776 | 372 (48) | 295 (38) | 19 (2) |
| E. Harerge | Kurfacale | 780 | 347 (44) | 263 (34) | 0 (0) |
| E. Shewa | Adami Tulu | 764 | 631 (83) | 233 (30) | 35 (5) |
| E. Shewa | Fentale | 790 | 218 (28) | 444 (56) | 16 (2) |
| E. Shewa | Lome | 987 | 691 (70) | 403 (41) | 21 (2) |
| E. Shewa | Adama | 784 | 490 (63) | 201 (26) | 33 (4) |
| E. Wellega | Wama Hagalo & BoneyaBushe | 775 | 498 (64) | 405 (52) | 12 (2) |
| E. Wellega | Diga | 780 | 608 (78) | 491 (63) | 39 (5) |
| E. Wellega | Gudaya Bila&Gubu Sayo | 778 | 663 (85) | 615 (79) | 50 (6) |
| E. Wellega | Guto Gida | 781 | 563 (72) | 504 (65) | 16 (2) |
| E. Wellega | Jima Arjo | 780 | 480 (62) | 435 (56) | 23 (3) |
| E. Wellega | Leka Dulecha | 781 | 587 (75) | 437 (56) | 37 (5) |
| E. Wellega | Nunu Kumba | 778 | 409 (53) | 388 (50) | 40 (5) |
| E. Wellega | Sasiga | 801 | 417 (52) | 499 (62) | 42 (5) |
| E. Wellega | Sibu Sire | 750 | 505 (67) | 507 (68) | 56 (7) |
| E. Wellega | Wayu Tuka | 779 | 636 (82) | 367 (47) | 24 (3) |
| Finfine Zuriya | Akaki | 831 | 564 (68) | 383 (46) | 2 (<1) |
| Guji | Anna Soraa | 780 | 590 (76) | 336 (43) | 7 (1) |
| Guji | Dama | 783 | 380 (49) | 497 (63) | 16 (2) |
| Guji | Bore | 780 | 444 (57) | 416 (53) | 18 (2) |
| Guji | Haro Wolabu | 785 | 269 (34) | 416 (53) | 4 (1) |
| Guji | Uraga | 785 | 470 (60) | 397 (51) | 41 (5) |
| Illu Aba bora | Metu | 899 | 698 (78) | 676 (75) | 14 (2) |
| Illu Aba bora | Alge Sachi | 906 | 565 (62) | 558 (62) | 2 (<1) |
| Illu Aba bora | Hurumu &Becho | 802 | 655 (82) | 588 (73) | 0 (0) |
| Illu Aba bora | Bilo Nopa | 967 | 821 (85) | 661 (68) | 1 (<1) |
| Illu Aba bora | Bure | 789 | 789 (100) | 589 (75) | 10 (1) |
| Illu Aba bora | Darimu | 795 | 662 (83) | 548 (69) | 3 (<1) |
| Illu Aba bora | Didu | 786 | 555 (71) | 599 (76) | 3 (<1) |
| Illu Aba bora | Doreni | 841 | 670 (80) | 467 (56) | 2 (<1) |
| Illu Aba bora | Yayo | 839 | 739 (88) | 600 (72) | 0 (0) |
| Jimma | Chora &Boter Tolay | 728 | 472 (65) | 369 (51) | 16 (2) |
| Jimma | Limmu Seka | 663 | 361 (54) | 256 (39) | 7 (1) |
| Jimma | Limmu Kossa | 869 | 606 (70) | 615 (71) | 19 (2) |
| Jimma | Deddo | 779 | 590 (76) | 404 (52) | 32 (4) |
| Jimma | Kersa | 780 | 573 (73) | 280 (36) | 77 (10) |
| Jimma | Mancho | 779 | 587 (75) | 221 (28) | 26 (3) |
| Jimma | Shabe Sombo | 779 | 572 (73) | 250 (32) | 20 (3) |
| Jimma | Sokoru | 777 | 592 (76) | 221 (28) | 21 (3) |
| Jimma | Tiro Afeta | 780 | 671 (86) | 283 (36) | 39 (5) |
| Kelem Wellega | Dale sedi | 780 | 764 (98) | 615 (79) | 32 (4) |
| Kelem Wellega | Seden Chanka & Dale Wabera | 780 | 697 (89) | 517 (66) | 53 (7) |
| Kelem Wellega | Lalo Kile | 780 | 712 (91) | 630 (81) | 57 (7) |
| North Shoa | Jidda & Abichugna | 790 | 660 (84) | 355 (45) | 12 (2) |
| North Shoa | Debre Libanos | 797 | 571 (72) | 455 (57) | 35 (4) |
| North Shoa | Degem | 797 | 373 (47) | 400 (50) | 5 (1) |
| North Shoa | Derra | 848 | 564 (67) | 542 (64) | 19 (2) |
| North Shoa | Girar Jarso | 786 | 756 (96) | 361 (46) | 1 (<1) |
| North Shoa | Kuyu | 784 | 350 (45) | 270 (34) | 2 (<1) |
| North Shoa | Were Jarso | 793 | 604 (76) | 400 (50) | 13 (2) |
| North Shoa | Wuchale | 798 | 736 (92) | 482 (60) | 28 (4) |
| North Shoa | Yaya Gulale | 788 | 528 (67) | 428 (54) | 0 (0) |
| S.W. Shewa | Ameya | 780 | 587 (75) | 399 (51) | 3 (<1) |
| S.W. Shewa | Becho | 781 | 644 (82) | 532 (68) | 8 (1) |
| S.W. Shewa | Dawo | 779 | 434 (56) | 496 (64) | 7 (1) |
| S.W. Shewa | Elu | 788 | 751 (95) | 581 (74) | 34 (4) |
| S.W. Shewa | Goro | 782 | 663 (85) | 431 (55) | 30 (4) |
| S.W. Shewa | Kersa Malima | 779 | 348 (45) | 217 (28) | 1 (<1) |
| S.W. Shewa | Seden Sodo R. | 777 | 621 (80) | 404 (52) | 6 (1) |
| S.W. Shewa | Sodo Dachi | 779 | 351 (45) | 149 (19) | 4 (1) |
| S.W. Shewa | Tole | 779 | 328 (42) | 438 (56) | 1 (<1) |
| S.W. Shewa | Woliso | 780 | 547 (70) | 328 (42) | 1 (<1) |
| S.W. Shewa | Wonchi | 780 | 443 (57) | 345 (44) | 8 (1) |
| W. Shewa | Ambo Zuria | 784 | 364 (46) | 461 (59) | 4 (1) |
| W. Shewa | Chobi | 780 | 192 (25) | 332 (43) | 5 (1) |
| W. Shewa | Dendi | 780 | 391 (50) | 414 (53) | 72 (9) |
| W. Shewa | Dire Inchini | 779 | 373 (48) | 401 (51) | 8 (1) |
| W. Shewa | Ejersa Lafo | 895 | 750 (84) | 636 (71) | 55 (6) |
| W. Shewa | Elfeta | 784 | 503 (64) | 417 (53) | 6 (1) |
| W. Shewa | Gindeberet | 780 | 529 (68) | 509 (65) | 98 (13) |
| W. Shewa | Jeldu | 780 | 406 (52) | 400 (51) | 11 (1) |
| W. Shewa | Jibat | 779 | 305 (39) | 427 (55) | 30 (4) |
| W. Shewa | Liben Jawi | 778 | 255 (33) | 502 (65) | 5 (<1) |
| W. Shewa | Nono | 782 | 588 (75) | 259 (33) | 34 (4) |
| W. Shewa | Toke Kutaye | 779 | 402 (52) | 442 (57) | 29 (4) |
| West Arsi | Arsi Negele | 788 | 637 (81) | 244 (31) | 51 (6) |
| West Arsi | Heben Arsi | 781 | 534 (68) | 465 (60) | 58 (7) |
| West Arsi | Adaba | 779 | 342 (44) | 212 (27) | 1 (<1) |
| West Arsi | Dodola | 779 | 337 (43) | 269 (35) | 3 (<1) |
| West Arsi | Gadeb Asasa | 779 | 467 (60) | 197 (25) | 32 (4) |
| West Arsi | Kofele | 780 | 349 (45) | 242 (31) | 3 (<1) |
| West Arsi | Kokosa | 781 | 338 (43) | 184 (24) | 3 (<1) |
| West Arsi | Kore | 780 | 400 (51) | 85 (11) | 1 (<1) |
| West Arsi | Nensebo | 781 | 343 (44) | 340 (44) | 6 (1) |
| West Arsi | Wondo | 780 | 745 (96) | 116 (15) | 26 (3) |
| West Wellega | Begi | 780 | 663 (85) | 670 (86) | 37 (5) |
| West Wellega | Yubdo & Homa | 779 | 738 (95) | 632 (81) | 19 (2) |
| West Wellega | Kiltu Kara | 781 | 704 (90) | 680 (87) | 48 (6) |
| Total | | 103,549 | 67,007 (65) | 49,790 (48) | 3,736(4) |


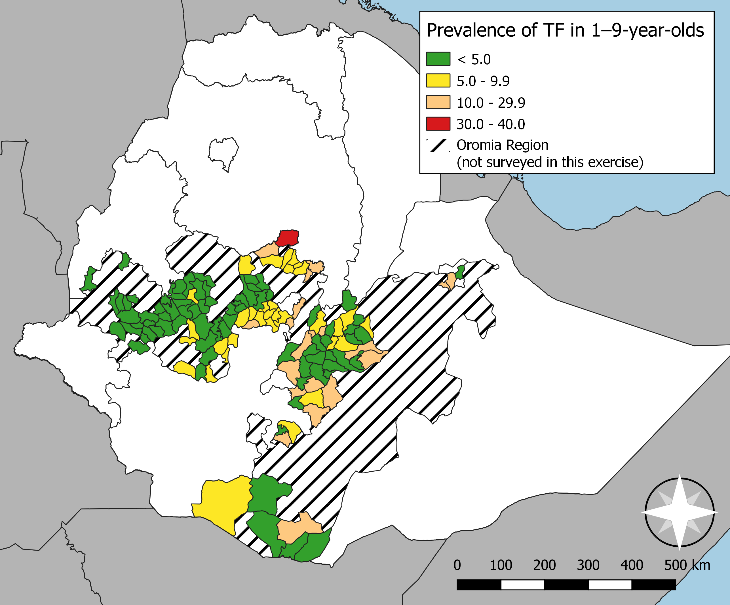

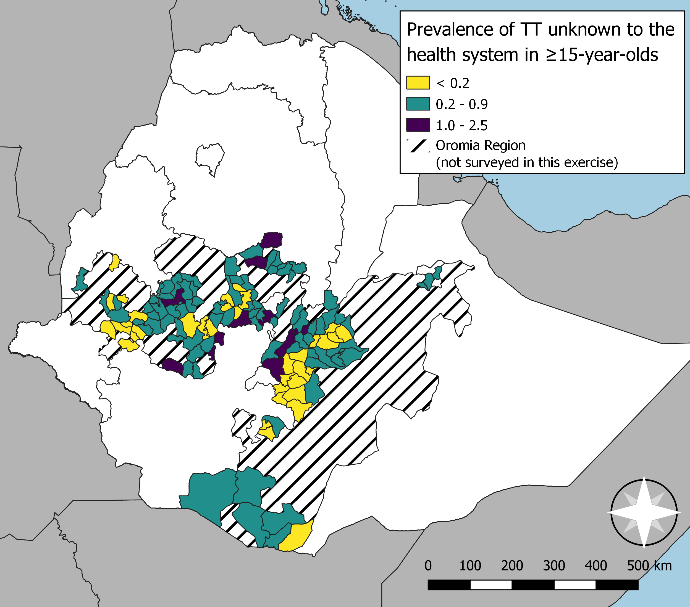


**Supplementary Figure 1.**Prevalence of trachomatous inflammation—follicular (TF) in 1–9-year-olds and trachomatous trichiasis (TT) unknown to the health system in ≥15-year-olds at trachoma impact surveys in Oromia Region, Ethiopia, February 2017−March 2020. TT was defined according to contemporary guidelines for each survey, which changed part-way through this survey series.^18^The boundaries and names shown and the designations used on this map do not imply the expression of any opinion whatsoever on the part of the authors, or the institutions with which they are affiliated, concerning the legal status of any country, territory, city or area or of its authorities, or concerning the delimitation of its frontiers or boundaries.
